# Emergency Department Frequentation and Unscheduled Visits of Liver Transplant Patients: Ten Years Experience in Tertiary Care Center, Saudi Arabia

**DOI:** 10.7759/cureus.46669

**Published:** 2023-10-08

**Authors:** Ahmad Aljumaa, Saad AlShathri, Jihad Aljumaa, Majd Alani, Hossam A Basha, Abdul Hadi F Afzal, Nayef Latta, Mohammed Almousallam, Saleh M Al-Yahri, Abdulrahman Alsulaiman, Farooq Pasha

**Affiliations:** 1 Emergency Medicine, Alfaisal University College of Medicine, Riyadh, SAU; 2 Emergency Department, King Faisal Specialist Hospital and Research Centre, Riyadh, SAU

**Keywords:** emergency department frequentation, emergency department visit rates, transplant recipient, living donor liver transplant-ldlt, liver transplantation

## Abstract

Introduction and objectives

Liver transplantation and its indications represent an increased burden on patients' health. This can be represented in a variety of ways, such as in emergency visits, unscheduled clinic visits, and unanticipated admissions. This study aims to analyze emergency department (ED) visits, the most common presenting complaints, and the outcomes of those visits.

Methods

A retrospective observational study was performed in which electronic medical records were reviewed for all patients who underwent liver transplantation and presented to the emergency department between October 2011 and October 2021. The following data were collected: demographics, comorbidities, liver transplant features, and emergency department visit data and outcomes. Recurrent visits were assessed and labeled as first, second, third, fourth, and fifth or more.

Results

A total of 699 patients and 5,225 visits were included in the analysis. Living donors accounted for 80% of all transplants. The mean post-operative length of stay was 22.6 ± 17.8. The majority of patients (74%) had at least one comorbidity, with diabetes (47%), hypertension (31%), and chronic kidney disease (CKD) (16%) being the commonest comorbidities; however, comorbidities were not associated with an increased risk of emergency department visits. Out of the 5,225 visits to the emergency department, 2,265 (41%) were within the first year. Emergency department visits in the first seven days after discharge amounted to 22% of total first visits. By 30 days, six months, and one year, they increased to 46%, 83%, and 91%, respectively. Living donor recipients had an average of 7.3 ED visits as compared to deceased donor recipients, who had an average of 8.4 ED visits. The most common presenting complaints were gastrointestinal (GI) symptoms (18%), infectious disease symptoms (9%), and respiratory symptoms (8%). There were a total of 296 patients who were readmitted at least once since discharge after liver transplantation.

Conclusion

The majority of first-time presentations to ED occurred in the first year post-transplant, marking this time period as critical for liver transplant patients. Our study also highlighted the continual presentations of liver transplant patients to the ED a few years post-transplant. This requires close scrutiny of the main causes of such presentations as well as comparison with other transplant cases to identify whether they are specific to liver transplants or not.

## Introduction

In 2021, more than 144,000 organs were transplanted worldwide, which represents an 11.3% increase compared to 2020. Liver transplants accounted for more than 24% of those transplants [1]. A liver transplant, like other organ transplants, is a complex procedure requiring painstaking preparation, specialized equipment, and advanced technical skills. A major factor that adds to the difficulty of liver transplant is the need to allocate the scarce donated livers to the increasing number of patients in need of and likely to benefit from this procedure - the recently included groups of acute alcoholic hepatitis, intrahepatic cholangiocarcinoma, and colorectal metastasis patients [2], as well as the previously included groups of patients with decompensated cirrhosis due to hepatitis B, hepatitis C, autoimmune hepatitis, primary biliary cirrhosis, and primary sclerosing cholangitis [3]. Other factors that contribute to the complicated nature of liver transplantation include the need for lifelong immunotherapy [4], and the associated surgical and medical complications [5-8]. With the continual rise of cirrhosis worldwide [9], the demand for liver transplants is expected to rise, as will the need to address all associated factors and complications. Liver transplantation constitutes one of the most studied topics in medical literature, with more than 48,400 articles containing the keywords "liver" and transplant" in their titles [10]. While liver transplant recipients can present to the clinic, imaging department, operation theater, and emergency department (ED), the ED represents one setting of great significance as it highlights the many complications and complaints that patients can present with. Though many studies have been conducted on liver transplant patients, as previously mentioned, very few articles have assessed their ED visits. A total of 10 articles within the existing body of literature on liver transplantation have specifically examined emergency visits. Among these works, two were review articles [6, 11], while two others did not primarily focus on liver transplantation [12, 13]. Thus, only six literary articles in all focused on emergency visits for liver transplant recipients. The earliest study done in 1998 described the literature regarding emergency visits in liver transplant patients as scant and highlighted the need for further studies that would help formulate guidelines for this subgroup of patients [11]. Although more than 25 years have passed, the literature remains as scant as it was decades ago. Even though there is considerable research on liver transplants in Saudi Arabia, no study has ever been done to assess liver transplant patients’ visits to ED, and until very recently, not one study has been conducted to assess solid organ transplant patients’ visits to ED. Our study aims to characterize liver transplant patients’ visits to the ED and identify any risk factors that contributed to those visits. Along with the very recent study assessing kidney transplant patients’ visits to the ED [14], this study will provide a better understanding and picture of the current status of organ transplant patients’ visits to the ED.

## Materials and methods

This was a retrospective observational study that was approved by the institutional board review of King Faisal Specialist Hospital and Research Center (KFSH&RC). All methodologies utilized in this research were conducted in adherence to the appropriate standards and regulations. Informed consent was waived by the IRB due to the retrospective nature of the study. 

Electronic medical records of all patients 18 years and above, who underwent liver transplantation in KFSH&RC and presented to the emergency department during October 2011 and October 2011 were reviewed. Patients who underwent other solid organ transplants and who were not following up in KFSH&RC were excluded from the study. 

To identify risk factors for emergency visits as well as characterize those visits, the following data were obtained from the electronic health record: demographics, comorbidities, liver transplant features (indications for transplant/coexisting liver disease, time since transplant, type of transplant, the relationship of donor to recipient, postoperative length of stay and complications of transplant); ED visit data (initial vital signs, reason for ED visit, emergency severity index level indicative of triage acuity, laboratory studies, consultation with liver transplant service, disposition of the patient after ED, evaluation in case of admission); and outcomes (in-hospital death, ward admission, total number of ED visits, frequency of ED visits, causes of hospital readmissions and their relative length of stay, number of days between liver transplantation and the first, second, third, fourth and fifth or more ED visit). Patients presenting symptoms will further be classified according to the involved organ system. 

A descriptive statistical analysis of the results was performed using SPSS (IBM Corp. Released 2017. IBM SPSS Statistics for Windows, Version 25.0. Armonk, NY: IBM Corp). Frequencies and percentages were used to express categorical variables and mean and median measures were used to express numerical variables. A significance level of p < 0.05 was used. 

## Results

Patient characteristics

A total of 699 patients were identified and included in the analysis. As described in Table 1, 80.5% (563/699) were living donor liver transplants. The post-operative length of stay after liver transplantation (LT) was 22.6 ± 17.8 days.

**Table 1:** Patient's Characteristics

Characteristics	No. (699)
Age	57.3±14.7 years
Sex	432 (61.8) males/267 (38.2) females
Living donor/deceased donor	563 (80.5%) living donors/ 136 (19.5%) deceased donors
Post-operative length of stay	22.6 ± 17.8 days

Comorbidities

Out of the 699 patients, 516 had at least one comorbidity. As described in Table 2, diabetes mellitus was the most common pre-existing comorbidity that was associated with liver transplant patients, followed by hypertension with 214 patients and chronic kidney disease (CKD) with 112. All CKD stages were involved in the analysis. The number of liver transplant patients who had coronary artery disease or dyslipidemia was 27 and 29, respectively, which is considered low compared to the population size.

**Table 2:** Patients' Comorbidities The most common presenting comorbidities in liver recipients presenting to the emergency department. DM: diabetes mellitus; HTN: hypertension; CKD: chronic kidney disease; CAD: coronary artery disease

Comorbidities	N (%)
DM	330 (47.2)
HTN	214 (30.6)
CKD	112 (16.0)
Dyslipidemia	29 (4.1)
CAD	27 (3.9)
None	183 (26.2)

Coexisting liver diseases

According to Table 3, viral hepatitis accounted for 40% of all coexisting liver conditions, with non-alcoholic steatohepatitis (NASH) accounting for 20.3% of the total, therefore highlighting the importance of those conditions and their association with end-stage liver disease and the need for transplantation. 

**Table 3:** Coexisting Liver Condition HCC: hepatocellular carcinoma; HCV: hepatitis C virus; NASH: non-alcoholic steatohepatitis; HBV: hepatitis B virus; AIH: autoimmune hepatitis. Prevalence of coexisting liver conditions in liver transplantation candidates.

Coexisting Liver Condition	N (%)
HCC	192 (27.5)
Alcoholic liver cirrhosis	14 (2.0)
HCV	145 (20.7)
NASH	142 (20.3)
HBV	135 (19.3)
AIH	80 (11.4)
Other	179 (25.6)

Causes of ED visits after transplant

There were a total of 5,225 ED visits after discharge post-liver transplantation. As shown in Tables 4, 5 respectively, the majority of ED visit causes were attributed to gastrointestinal causes, constituting 18% of the total visits causes, with abdominal pain being the leading cause in that category with 789 visits, diarrhea or constipation being the second leading cause with 333 visits, and jaundice being the 3rd leading cause in that category with 24 visits. This was followed by fever, which accounted for 477 (9%) of the total causes of visits. Respiratory causes were the third most common cause of ED visits, making up 8% of the total visits, with shortness of breath being the leading symptom in that category with 249 visits and upper respiratory tract symptoms (flu) being the second leading cause in that category with 92 visits. Musculoskeletal causes were the fourth most common cause of ED visits, accounting for 5% of the total visits, with back pain being the leading symptom in that category with 160 visits. Renal causes were the fifth most common symptom of ED visits, constituting 4% of the total visits, with dysuria and hematuria being the leading symptoms in that category with 122 visits and flank pain being the second leading symptom in that category with 90 visits. Central nervous system causes were the sixth most common cause of ED visits, accounting for 4% of the total visits, with dizziness being the leading symptom in that category with 111 visits. Dermatological symptoms were the seventh most common cause of ED visits, making up 3% of the total visits, with pruritus being the leading symptom in that category with 58 visits and skin reactions or rash being the second leading complaint in that category with 28 visits. Abnormal lab-related causes were the eighth most common symptom of ED visits, causing 3% of the total visits, with abnormal liver function tests being the leading cause in that category with 55 visits. Cardiovascular complaints were the least contributing causes of ED visits, making up 1% of the total visits, with chest pain being the leading symptom in that category with 144 visits.

**Table 4:** Causes of ED Visits After Transplant (System Based) Most commonly involved organ systems in liver recipients presenting to the emergency department.

ER Visit Reason	N (%)
Gastrointestinal	950 (18.2)
Infectious	477 (9.1)
Respiratory	400 (7.7)
Musculoskeletal	243 (4.7)
Renal	196 (3.8)
Neurological	183 (3.5)
Skin	164 (3.1)
Labs	132 (2.5)
Cardiac	40 (0.8)
Other	2440 (46.7)

**Table 5:** Causes of ED Visits after Transplant (Symptom Based) The most commonly presenting symptoms in liver recipients presenting to the emergency department. RUQ: right upper quadrant; SOB: shortness of breath; URTI: upper respiratory tract infection; LFT: liver function test.

ER Visit Reason	No. (5225)
Abdominal pain, RUQ pain, or Epigastric pain	789 (35.9)
Diarrhea or Constipation	333 (15.1)
SOB or Dyspnea	249 (11.3)
Back pain	160 (7.3)
Chest pain	144 (6.5)
Dysuria or UTI or hematuria	122 (5.5)
Dizziness	111 (5.0)
Flu symptoms or URTI	92 (4.2)
Flank pain	90 (4.1)
Pruritis or Itchiness	58 (2.6)
Abnormal LFT or High LFT	55 (2.5)
Skin reaction or Rash	28 (1.3)
Jaundice	24 (1.1)

Table 6 provides an analysis of the ED visits. The distribution is based on the number of visits and the time period. Our study assessed both early and late presentations to the ER, which was not conducted by any previous studies known to our knowledge. The ED visits continued even after three years of transplantation. Our study covered a period of 10 years. As seen in the table, there were 1353 visits to the ED after the first three years of the transplant, highlighting the continual visits to the ED throughout the years and their high frequency.

**Table 6:** Number of Emergency Department Visits after Liver Transplantation Data are presented as numbers (%).

ED Visits	<7 days (n=78)	<30 days (n=306)	<6 months (1067)	<1 year (n=765)	<2 years (n=968)	<3 years (n=588)	>3 years (n=1353)
1 (n=699)	153 (22)	168 (24)	259 37)	56 (8)	38 (6)	9 (1)	16 (2)
2 (n=607)	22 (4)	91 (15)	265 (44)	106 (18)	62 (10)	26 (4)	35 (25)
3 (n=496)	2 (0.4)	29 (6)	204 (4)	107 (22)	69 (14)	32 (6)	53 (11)
4 (n=437)	1 (0.2)	15 (3)	124 (28)	99 (23)	93 (23)	43 (10)	62 (14)
5 (n=2986)	0	3 (0.1)	215 (7)	397 (13)	706 (24)	478 (16)	1187 (40)

Causes of readmission based on presenting ED symptoms: There were a total of 296 patients who got readmitted at least once since discharge after liver transplantation. As indicated by Table 7, the leading causes for the first, second, third, and fourth readmissions were the same. Out of the 296 first readmissions, the leading cause was abdominal pain with 113 readmissions followed by diarrhea or constipation with 43 readmissions. Out of the 288-second readmissions, the leading cause was abdominal pain with 104 readmissions followed by diarrhea or constipation with 39 readmissions. Out of the 223 third readmissions, the leading cause was abdominal pain with 78 readmissions followed by diarrhea or constipation with 27 readmissions. Out of the 189 fourth readmissions, the leading cause was abdominal pain with 64 readmissions followed by diarrhea or constipation with 29 readmissions. There were a total of 996 readmissions. Patients with abnormal LFTs, shortness of breath, and jaundice experienced a prolonged hospital stay of an average of 12 days, eight days, and eight days, respectively. Skin reactions, flu symptoms, and chest pain were found to be associated with some of the shortest hospital stays, averaging just two days, four days, and four days, respectively.

**Table 7:** Causes of Hospital Readmission The causes of subsequent hospital readmissions post-emergency visits and their frequencies in liver recipients.

1^st^ readmission *(N= 296)*	2^nd^ readmission *(N= 288)*	3^rd^ readmission *(N= 223)*	4^th^ readmission *(N= 189)*
Cause of readmission (n,%)			
Abdominal pain, RUQ pain, or Epigastric pain (113, 16.2)	Abdominal pain, RUQ pain, or Epigastric pain (104, 17.1)	Abdominal pain, RUQ pain, or Epigastric pain (78, 15.2)	Abdominal pain, RUQ pain, or Epigastric pain (64, 12.5)
Diarrhea or Constipation (43, 6.2)	Diarrhea or Constipation (39, 6.4)	Diarrhea or Constipation (27, 5.3)	Diarrhea or Constipation (29, 5.6)
SOB or Dyspnea (25, 3.6)	SOB or Dyspnea (28, 4.6)	SOB or Dyspnea (25, 4.9)	SOB or Dyspnea (25, 4.9)
Dizziness (22, 3.2)	Chest pain (19, 3.1)	Back pain (24, 4.7)	Back pain (14, 3.2)
Back pain (19, 2.7)	Dizziness (16, 2.6)	Chest pain (15, 2.9)	Dizziness (14, 2.7)
Chest pain (14, 2.0)	Flu symptoms or URTI (15,2.5)	Flu symptoms or URTI (13, 2.5)	Flank pain (9, 1.8)
Flu symptoms or URTI (13, 1.9)	Back pain (15, 2.5)	Dizziness (9, 1.8)	Flu symptoms or URTI (8, 1.8)
Dysuria or UTI or hematuria (11,1.6)	Dysuria or UTI or hematuria (12,2.0)	Dysuria or UTI or hematuria (8, 1.6)	Dysuria or UTI or hematuria (7, 1.4)
Skin reaction or Rash (11, 1.6)	Flank pain (11, 1.8)	Pruritis or Itchiness (7, 1.4)	LFT (5, 1.0)
Flank pain (9, 1.3)	LFT (10, 1.6)	LFT (6, 1.2)	Chest pain (5, 1.0)
LFT (9, 1.3)	Pruritis or Itchiness (8, 1.3)	Skin reaction or Rash (5, 1.0)	Skin reaction or Rash (3, 0.7)
Pruritis or Itchiness (4, 0.6)	Jaundice (5, 0.8)	Jaundice (3, 0.6)	Jaundice (3, 0.6)
Jaundice (3, 0.4)	Skin reaction or Rash (6, 1.1)	Flank pain (3, 0.6)	Pruritis or Itchiness (3, 0.6)

## Discussion

As mentioned earlier, there has been an increase in the liver transplant subgroup of patients in recent years. The study conducted represents one of the studies with the largest number of liver transplant patients. Compared to other studies done over the years in different countries with sample sizes of 56, 332, and 430 liver transplant patients [15-17], our study included 699 patients in its analysis with a total of 5225 emergency visits. This highlights the increased number of patients undergoing liver transplants and the necessity of further understanding their needs and emergencies. While the studies referenced earlier focused on the early ED visits post-liver transplant, our study assessed both early and late ED presentations. ED visits in the first seven days after discharge amounted to 22% of total first visits. By 30 days, six months, and one year, they increased to 46%, 83%, and 91%, respectively. This indicates that the first year and especially the first six months are a critical period for transplant patients, as the likelihood of a first emergency visit occurring within the first year of discharge is as high as 91%.

Another result that highlights the importance of this period after transplant is the total number of visits during the first year. Out of 5225 visits to the ED, 2265 (41%) were within the first year, and 1,501 (66%) of those visits were within the first six months. Two similar studies had shown similar results, with ED visits within the first six months accounting for 60% of total visits within the first year [14,16]. The study conducted in South Korea was more similar to this study’s methodology, with visits three years post-live transplant being assessed. Taking only the first three years into consideration, first-year ED presentations accounted for 59% of total visits in those years, which is comparable to the 64% in the Korean study [17]. Compared to previously mentioned studies, our study assessed the visits of patients after three years of discharge and found the visits to be as high as 26% of all visits assessed. This highlights the continual visits of liver recipients to the ED, even after two years of discharge. Further studies are needed to assess those visits in comparison with the general population. Living donor transplants in our study comprised 80.5% of total transplants, consistent with transplants done in South Korea [17], as compared to transplants done in the US, in which living donor transplants were as low as 2.3% of total transplants [18]. Although this is the current status in the US, one study published in 2008 found a percentage of living donors as high as 44.4%. This accentuates the importance of carefully comparing data from different countries and timelines.

Living donor recipients had an average of 7.3 ED visits as compared to deceased donor recipients, who had an average of 8.4 ED visits. There was no statistical significance when it came to the source of the organ (P-value 0.326). While the literature available contains a few articles assessing emergency visits of liver recipients, only one study assessed associated comorbidities, in which 44% of the patients had at least one comorbidity, with diabetes, hypertension, and coronary heart disease being the commonest ones, respectively [16]. In our study, 516 patients (74%) had at least one comorbidity, with diabetes (47%), hypertension (31%), and CKD (16%) being the commonest comorbidities. Comorbidities were not found to be associated with an increased risk of ED visits (p-value = 621). The most common presenting complaints were GI symptoms (18%), infectious disease symptoms (9%), and respiratory symptoms (8%). These results were similar to those of the two studies done in South Korea and the US [13, 17]. Contrary to our results, two other studies had different outcomes, with one study having infectious, gastrointestinal, and musculoskeletal symptoms as the most common presenting complaints [16], and the other study having electrolyte imbalance, gastrointestinal symptoms, and infectious symptoms as the most common presenting complaints [16].

Our study had several limitations. The first was the retrospective nature of the study, in which all retrospective biases could apply. However, the high-quality data input and collection, as well as the consistent use of the same electronic system throughout the study period, provided highly reliable data. Considering our hospital is the main transplant center in Saudi Arabia, many patients were residents of another city other than where the hospital is located. Thus, not all ED visits might have been documented. Patients might have been presenting to primary care centers as well for minor complications instead of the tertiary center which adds another limitation. The study did not include all liver transplant patients, so no comparison could be made between those who presented to the ED and those who did not. The high number of living donors can present a barrier to comparison with studies done in other countries where deceased donor transplants are the most common.

## Conclusions

The majority of first-time presentations to ED occurred in the first year post-transplant, marking this time period as critical for liver transplant patients. Our study also highlighted the continual presentations of liver transplant patients to the ED a few years post-transplant. This required close scrutiny of the main causes of such presentations as well as comparison with other transplant cases to identify whether they were specific to liver transplants or not. Although we have verified and documented the number of hospital readmissions post-liver transplant along with the most common presenting complaints, additional studies are needed to assess the outcome of each admission and the prognostic value of such readmissions. Further in-depth reviews are required to compare the features of patients who present to the ED with those who do not in the first year, as well as to compare such patients’ odds of visiting the ED compared to the general population. Our study opens the door for further prospective research and additional studies aiming to assess the improvement of healthcare for this particular subgroup of patients.
